# When Breast Cancer Hides Elsewhere: Invasive Lobular Carcinoma Presenting as Gallbladder Disease

**DOI:** 10.7759/cureus.108440

**Published:** 2026-05-07

**Authors:** Erica B Kingdon, Kimlan H Phan, Megan M Woods, Evan Neuls

**Affiliations:** 1 College of Medicine, A.T. Still University, Kirksville College of Osteopathic Medicine, Kirksville, USA; 2 College of Medicine, University of Kansas School of Medicine, Kansas City, USA; 3 Surgical Services, Western Missouri Medical Center, Warrensburg, USA

**Keywords:** breast cancer detection, breast cancer genetics, breast cancer metastasis, gall blader disease, invasive lobular carcinoma of the breast, surgical case report

## Abstract

Invasive lobular carcinoma is a type of breast cancer that is distinctive in morphology. Due to this, invasive lobular carcinoma often proves to be difficult for clinicians to detect and treat before metastasis occurs. While invasive lobular carcinoma shares common metastasis locations with other breast cancers, such as infiltrating ductal carcinoma, invasive lobular carcinoma is notorious for presenting in abnormal sites. This report will introduce an atypical case of invasive lobular carcinoma first discovered in the gallbladder. A 58-year-old female presented to the emergency department with a one-week history of right upper quadrant pain. After an ultrasound revealed multiple stones within the gallbladder, she underwent an elective cholecystectomy. Pathology showed findings indicative of invasive lobular carcinoma of the breast. A follow-up mammogram and ultrasound showed no abnormalities of the breasts; however, a PET/CT scan revealed an area of abnormal metabolic activity in the right breast. Subsequent genetic testing unveiled a missense mutation in the CDH1 gene. We will discuss the atypical findings of this presentation of invasive lobular carcinoma, specific genetic markers associated with this type of malignancy, and the unique features of this type of cancer that render early detection difficult.

## Introduction

In the United States, approximately 1 out of 8 women will be diagnosed with invasive breast cancer, and of these diagnoses, 10%-15% of them will have invasive lobular carcinoma (ILC) [1,2]. While annual mammograms are the recommended screening test for breast cancer, ILC in particular is evasive and can be difficult to detect [1], which can result in a misleading normal mammogram. This is due to the unique characterization of ILC: single-file cellar infiltration that fails to disrupt the natural architecture of surrounding tissue [1]. Similar to infiltrating ductal carcinoma (IDC), potential sites of metastasis of invasive lobular carcinoma include the liver, lung, and lymph nodes. However, invasive lobular carcinoma has additional metastatic potential to spread to abnormal sites such as bone, the gastrointestinal tract, adnexa, and peritoneum [3]. Although ILC is often associated with good prognostic outcomes due to its typically presenting as estrogen- and progesterone-receptor positive, the staggering metastatic potential of ILC frequently leads to diagnosis after metastasis has already occurred [1]. We present an unusual case of ILC diagnosis after first discovering it in the gallbladder after an elective cholecystectomy. This work was previously presented in poster format at the Missouri Association of Osteopathic Physicians & Surgeons 2026 Missouri Osteopathic Annual Convention on April 25, 2026.

## Case presentation

A 58-year-old previously healthy woman presented to the emergency department with a one-week history of progressive right upper quadrant pain and occasional nausea. She did not have any fevers, vomiting, or right shoulder pain. Laboratory values were adequate, with liver function tests presenting as the following: ALT 16 U/L (reference range: 0-55 U/L), AST 21 U/L (reference range: 5-34 U/L), ALP 87 U/L (reference range: 40-150 U/L), and total bilirubin 0.3 mg/dL (reference range: 0.19-1.16 mg/dL). An ultrasound revealed cholelithiasis (Figure 1A), 0.5 cm of gallbladder wall thickening (Figure 1A), hepatomegaly (Figure 1B), and steatosis (Figure 1C). Assuming a diagnosis of acute cholecystitis, a laparoscopic cholecystectomy was performed on hospital day two.

**Figure 1:**
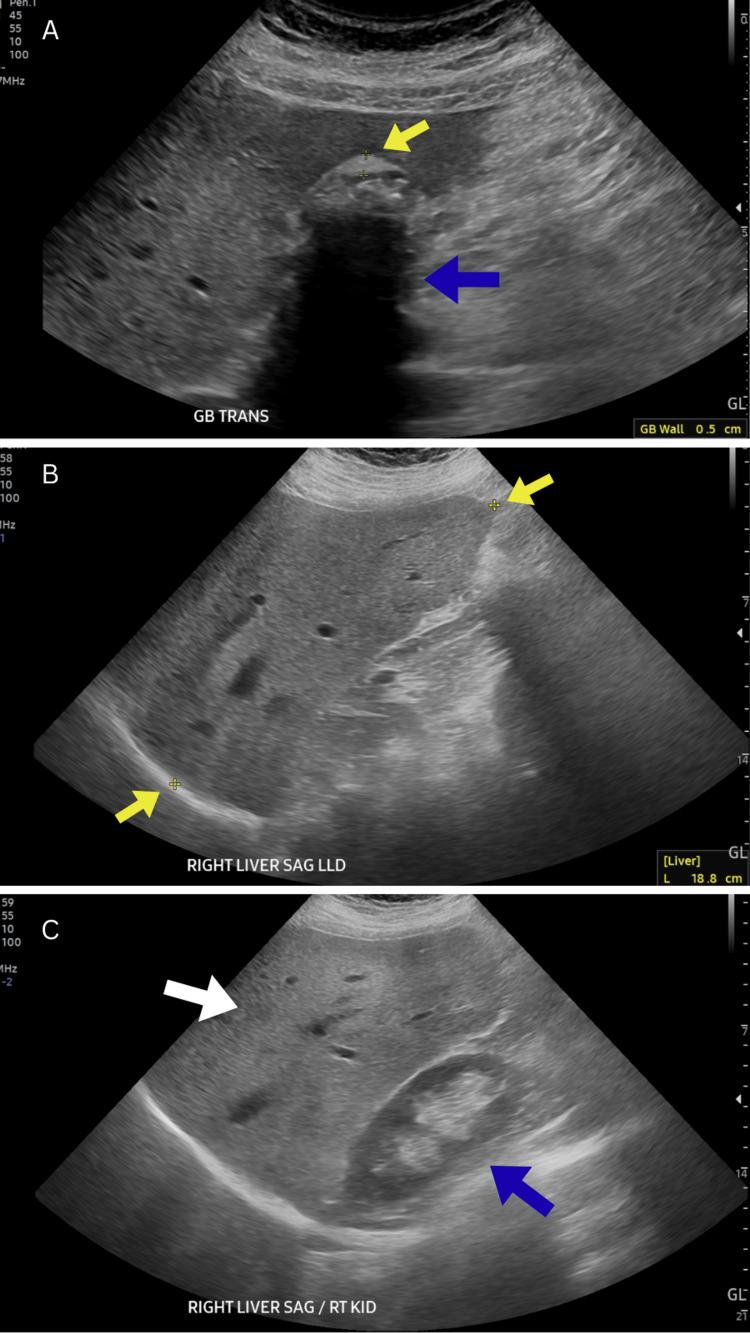
Ultrasound images of the gallbladder and liver obtained in the emergency department. A) 0.5 cm of gallbladder wall thickening was measured (yellow arrow), and multiple stones were indicated by the shadowing (blue arrow). B) Hepatomegaly as indicated by the 18.8 cm measurement of the liver (yellow arrows); C) Steatosis of the liver (white arrow) can be identified by the hyperechoic liver next to the right kidney (blue arrow).

During the procedure, chronic inflammatory changes in the gallbladder were noted, as well as innumerable stones within (Figure 2A). Dense adhesions of the omentum to the gallbladder were found (Figure 2B), and a biopsy of the abnormal omentum was obtained. An intraoperative esophagogastroduodenoscopy (EGD) was also performed at this time due to plaque-like lesions found on the stomach and the unusual presentation of the omentum (Figure 2C). Moderate erythematous inflammation of the cardia of the stomach and gastric body was visualized (Figure 2D); however, no bleeding or ulceration was seen. Biopsies of the gastric body, omentum, and gallbladder were sent to pathology for analysis.

**Figure 2:**
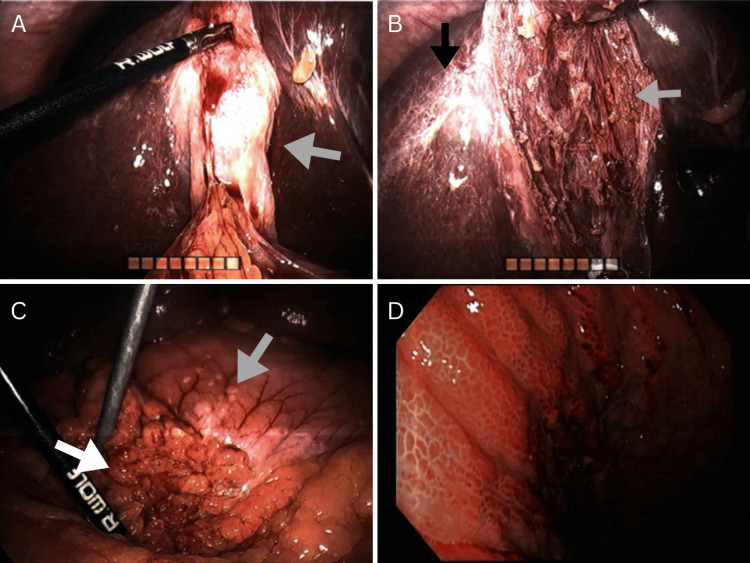
Images taken during cholecystectomy and esophagogastroduodenoscopy. A) Chronic inflammatory changes seen on the outside of the gallbladder (gray arrow); B) Steatosis of the liver (black arrow) and remnants of the dense adhesions of omentum that engulfed the gallbladder (gray arrow); C) Abnormal nodularity of omentum (white arrow) and plaque-like lesions on the stomach (gray arrow); D) Inflammation of the gastric body seen on EGD.

At pathology, the provided specimen showed prominent single-cell infiltration and Indian filing within the walls of the gallbladder (Figures 3A, 3B), which are defining characteristics of ILC [1]. Additionally, the specimen also exhibited expected immunohistochemical studies for ILC: negative for e-cadherin and HER2, and positive for keratin OSCAR (Figure 4A), estrogen receptor (Figure 4B), progesterone receptor, cytokeratin-7, and GATA-3 (Figure 4C). PAX8 and cytokeratin-20 were also negative. Pan-keratin staining of the gastric biopsy showed gastric epithelia with no evidence of metastatic carcinoma.

**Figure 3:**
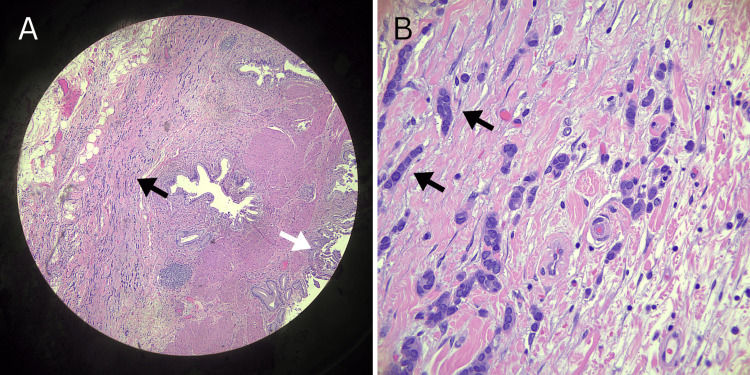
Gallbladder pathology. A) Complete section of gallbladder wall with the mucosa intact (white arrow) and tumor cells invading the tunica muscularis (black arrow); B) Single-file infiltration of tumor cells in the tunica muscularis (black arrows).

**Figure 4:**
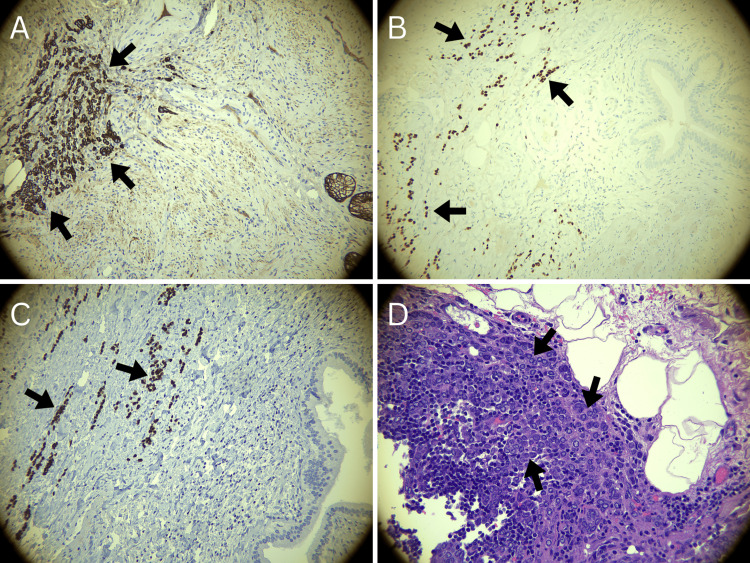
Invasive lobular carcinoma pathology stains and metastasis to sentinel lymph nodes. A) Tumor cells staining positive for keratin-OSCAR (black arrows); B) Tumor cells staining positive for estrogen receptor (black arrows); C) Tumor cells staining positive for GATA-3 (black arrows); D) Section of sentinel lymph node of the gallbladder containing ILC metastatic tumor cells (black arrows).

The pathology report revealed ILC diffusely involving the serosal surface of the gallbladder wall, fat around the cystic duct, and metastasis to the periductal (sentinel) lymph node (Figure 4D). Chronic cholecystitis with cholesterolosis and cholelithiasis was also noted. The biopsy of the omentum also revealed ILC. The stomach biopsy showed chronic antral gastritis with no evidence of metastatic ILC.

The patient presented to the surgery clinic on postoperative day nine to discuss the next steps. A breast examination was performed and revealed no breast pain, nipple discharge, or palpable lumps in either breast. However, an enlarged lymph node in the left axilla was found. The patient was unsure of the date of her last mammogram, but one was conducted shortly thereafter and revealed no abnormal breast tissue or suspicious axillary lymph nodes. An ultrasound was performed at this time, but no irregularities were noted on this type of imaging either. A PET/CT scan revealed an area measuring 1.5 cm demonstrating abnormal metabolic activity in the outer aspect of the right breast (Figure 5A) and metastasis to multiple bony structures that are consistent with diffuse bony metastatic disease (Figure 5B, 5C). An MRI without contrast of the abdomen, pelvis, and brain was performed, and further revealed bony metastasis but demonstrated no other abnormalities. Contrast could not be used due to the patient’s allergy to contrast dye. Images of the MRI were unable to be obtained from an outside institution due to logistical limitations; therefore, representative images of the MRI can not be included in this report. Additional blood tests revealed the metastatic breast cancer markers CA 15-3 LC and CA 27.29 LC to be 406 U/mL (reference range: <30 U/mL) and 444 U/mL (reference range: <38 U/mL), respectively, indicating advanced metastatic breast disease. Genetic testing was promptly ordered and found a missense mutation in the CDH1 gene. Oncology officially diagnosed the patient with stage IV invasive lobular carcinoma with metastasis to the bone. She has been started on denosumab and is currently enrolled in a clinical trial for endocrine therapy.

**Figure 5:**
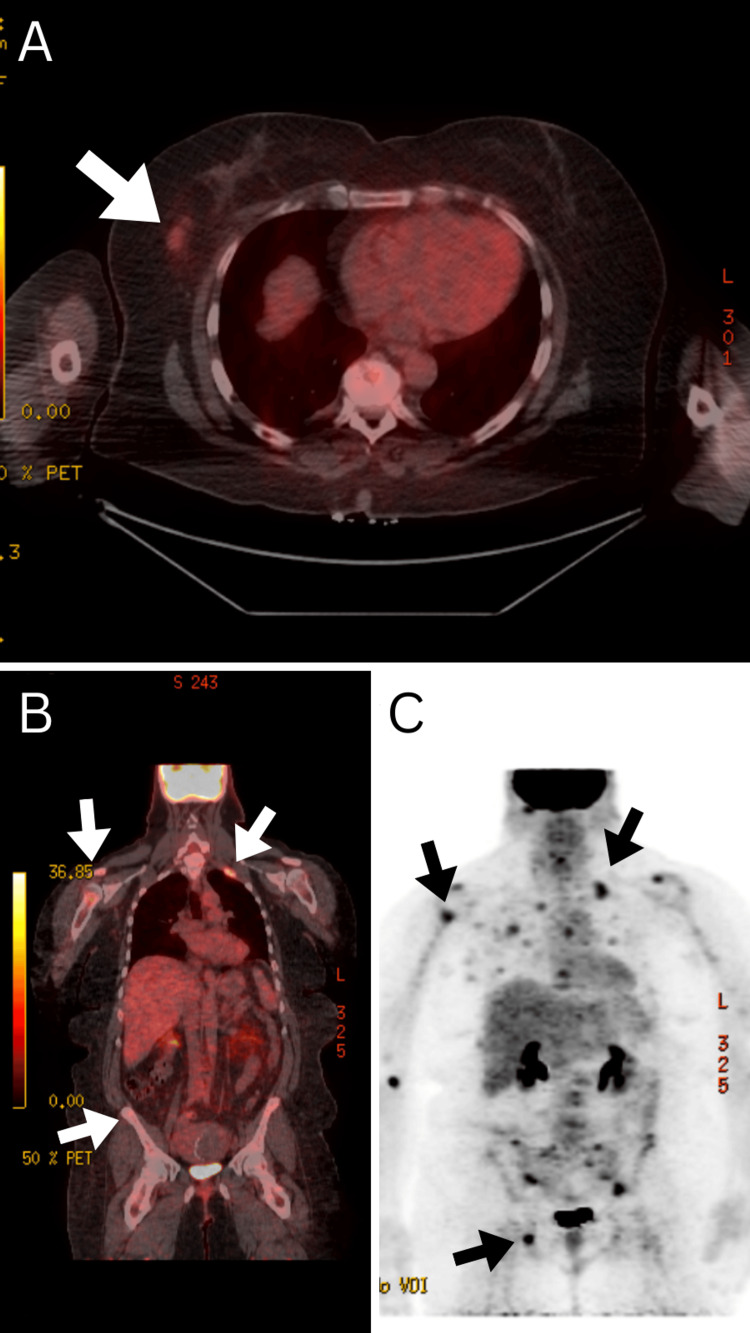
PET and PET/CT scans. A) PET/CT scan showing the 1.5 cm area of abnormal metabolic activity in the right breast (white arrow); B) PET/CT scan highlighting abnormal metabolic activity and bony metastasis (white arrows); C) PET scan showing areas of bony metastasis (black arrows).

## Discussion

Because of the unique pathological morphology of ILC, physical lumps and bumps may not be exposed on a breast exam, mammogram, or breast ultrasound. Failure to identify ILC during annual mammograms can lead to metastasis and a higher likelihood of mortality from the carcinoma. In our patient’s case, we saw that she had an unremarkable mammogram and ultrasound after the diagnosis of metastatic ILC. This coincides with the sensitivity of mammograms and ultrasounds for diagnosing ILC, which ranges from 34% to 92% and 68% to 92%, respectively [4]. This begs the question: how can physicians identify elusive cases of ILC before metastasis occurs?

While annual screening mammograms have proven to reduce the mortality of breast cancer, there are additional factors that should be considered when handling a patient who could potentially have breast cancer. A thorough family history should always be recorded, as 15% of breast cancer diagnoses are associated with having relatives with a breast cancer diagnosis as well [5]. In patients with a positive family history, genetic testing should be considered based on factors such as the degree of relative(s), age at diagnosis, and type of breast cancer [5]. Additionally, practitioners should always ask the patient's age at menarche, age at first birth, age at menopause (if applicable), and if the patient has ever received hormone replacement therapy. These factors assist in clarifying the risk patients may have for developing breast cancer. For example, ILC typically presents in patients who had early menarche, late menopause, late age at first birth, and those who have a history of hormone replacement therapy, as these factors are associated with longer hormone exposure [6]. Patients with personal or family histories consistent with these factors may warrant additional screening. All patients should be educated to be vigilant for any breast changes they may have noticed, such as breast size alterations, nipple shape modifications, or nipple dimpling [7]. These factors may seem insignificant to patients, but they could be clinical clues to help diagnose breast cancer and could provide substantial evidence for further work-up of the elusive ILC. In relation to this patient’s case, she did not exhibit any of these clinical symptoms. She reported that her age of menarche was 12, age of first birth was 25, and age of menopause was 51. Her only history of hormone use was levothyroxine, and she had no family history of breast cancer. These ages are all within the average ranges for these factors, and her lack of menopausal hormone therapy does not align with the typical ILC patient, making her case even more of an anomaly.

There are many genetic mutations associated with breast cancer, some of the most notable being BRCA1 and BRCA2. In the case of ILC, one of the most interesting mutations is CDH1. This gene is associated with the adhesion protein e-cadherin, which is diagnostically absent in ILC [1,6], as is seen in our patient. In females that carry the CDH1 mutation, about 50% are expected to develop ILC and therefore must be monitored with regular MRIs and mammograms [6]. For our patient, genetic testing revealed an autosomal dominant heterozygous missense mutation on the CDH1 gene at c.1375G>A (p.Val459Met). This specific mutation is reported to have uncertain significance; however, an algorithm (PolyPhen-2) used to predict the effect of missense changes on protein function and structure suggests that this specific mutation is likely to be disruptive. Though this missense mutation has uncertain significance due to its rarity, there is one other case in literature that reports this alteration. A case report discussed a 48-year-old female who was diagnosed with bilateral ovarian serous borderline tumors, and she was also found to have the CDH1 c.1375G>A mutation [8]. Another interesting detail that further connects these two case reports lies in family history. Whereas our patient reported no family history of breast cancer, the patient with the ovarian malignancy reported that she had a family history of maternal ovarian cancer at age 60 and two maternal aunts with breast cancer [8]. While additional research and evidence are needed to confirm the specific implications of this mutation, these findings suggest that there could potentially be a correlation between the CDH1 c.1375G>A mutation and breast and ovarian cancer.

Additionally, CDH1 mutations are also associated with diffuse gastric cancer [6]. Specifically, there are certain pathogenic variants of CDH1 that are associated with Diffuse Gastric and Lobular Breast Cancer Syndrome (DGLBCS) [9]. This syndrome predisposes both sexes to diffuse gastric cancer and increases the risk of female patients developing ILC [9]. The CDH1 c.1375G>A missense mutation found in our patient is of uncertain significance and has not been linked to DGLBCS previously. This aligns with the results of our patient’s gastric biopsy, which stained negative for ILC or other malignancies. However, our patient’s CDH1 mutation may provide insight on why there were some abnormalities visualized on EGD.

By the time of diagnosis, two-thirds of ILC cases have already metastasized [7]. While ILC typically has good prognostic features and a favorable response to endocrine-based treatment due to the presence of estrogen and progesterone receptors, progression of metastases may hinder treatment outcomes. This may explain why multiple studies have found that ILC has similar or worse longitudinal outcomes compared to invasive carcinoma of no special type (IC-NST) [1]. Additionally, the unique sites of metastasis likely have an impact on long-term survival. Although ILC shares sites of metastasis with IDC, such as the liver, lymph nodes, and lungs, ILC also has some abnormal sites of metastasis. Bone, gastrointestinal tract, gynecological, and peritoneal metastases are not uncommon for ILC [3,10]. Metastases to these locations are not completely understood, but they have been thought to be due to the architecture of ILC. The lack of e-cadherin causes abnormal cell-to-cell connectivity, which results in the diagnostic single-file pathology of ILC and the absence of breast nodules in patients [1,4]. This loss of e-cadherin has been hypothesized to contribute to the abnormal metastatic locations of ILC [1,10].

Regarding our patient, the gallbladder is a very rare site of metastasis for ILC. There have been fewer than 25 reported cases of metastatic breast cancer in the gallbladder in the literature [11]. Even more noteworthy is the fact that a literature review identified only two prior cases of ILC incidentally discovered in the gallbladder following cholecystectomy (references [11] and [12]). Because of the single-file nature of ILC, it is understandable that there was no detectable tumor mass within the gallbladder; rather, the carcinoma likely invaded the walls of the gallbladder due to the lack of e-cadherin. This is the probable cause of our patient’s cholecystitis-like symptoms when she first presented to the emergency department.

## Conclusions

ILC is notorious for metastasizing to abnormal locations and proves itself elusive in nature. We present an astonishing case of metastatic ILC first discovered in the gallbladder after a clinical mimicry of acute cholecystitis. The purpose of this report is not only to publicize a very rare case, but also to highlight the inimitable ILC and its stealthy disposition that can make diagnosis so challenging. Because of this, physicians should remain vigilant for evasive breast cancer even after a normal mammogram, especially in patients with abnormal breast symptoms or a family history of breast cancer. Additional research is needed to fully understand the nature of ILC and how to more accurately diagnose this carcinoma before metastasis occurs.
